# Uncovering a high-performance bio-mimetic cellular structure from trabecular bone

**DOI:** 10.1038/s41598-020-70536-7

**Published:** 2020-08-28

**Authors:** Abdallah Ghazlan, Tuan Ngo, Tuan Nguyen, Steven Linforth, Tu Van Le

**Affiliations:** Department of Infrastructure Engineering, The University of Melbourne, Parkville, VIC 3010 Australia

**Keywords:** Engineering, Civil engineering

## Abstract

The complex cellular structure of trabecular bone possesses lightweight and superior energy absorption capabilities. By mimicking this novel high-performance structure, engineered cellular structures can be advanced into a new generation of protective systems. The goal of this research is to develop an analytical framework for predicting the critical buckling load, Young’s modulus and energy absorption of a 3D printed bone-like cellular structure. This is achieved by conducting extensive analytical simulations of the bone-inspired unit cell in parallel to traverse every possible combination of its key design parameters. The analytical framework is validated using experimental data and used to evolve the most optimal cellular structure, with the maximum energy absorption as the key performance criterion. The design charts developed in this work can be used to guide the development of a futuristic engineered cellular structure with superior performance and protective capabilities against extreme loads.

## Introduction

Natural and man-made hazards such as blast^1,2^ and impact^3,4^ have claimed millions of lives and caused significant damage to structures. Engineered cellular structures are at the forefront of innovative research for extreme loading applications (e.g., protective structures and crashworthiness), due to their lightweight and superior energy absorption capabilities^5,6^. There is significant potential to advance these structures into a new generation of futuristic protective systems by mimicking cellular architectures found in nature^7–9^. In particular, bone has optimised its intricate structure through complex evolutionary processes to minimise weight, enhance mobility and meet the cyclic loading demands of the human body^9–12^. It has long been speculated by researchers that the key to the excellent mechanical properties of bone is hidden in the structural features of its well-organised cellular core, which is composed of organised networks of interconnected trabeculae^13,14^. Although trabecular bone is not primarily designed to absorb energy, it has been shown to possess unique structural characteristics that facilitate excellent energy absorption^15–17^. Despite that other biological cellular structures such as beetle elytra^7^ and porcupine quills^15,18,19^ have been reported to exhibit lightweight, buckling resilience and energy absorption characteristics through mechanical testing, investigations on biomimetic cellular structures for energy absorption applications are generally quite limited. Ghazlan et al.^20^ investigated a cellular structure inspired by trabecular bone under compression and demonstrated its high energy absorption over hexagonal and re-entrant structures. Zhang et al.^7^ fabricated a sandwich plate inspired by beetle elytra and demonstrated marked improvements in energy absorption over a traditional honeycomb structure under compression. Du et al.^21^ also fabricated a lattice structure inspired by beetle elytra and reported a high energy absorption and bearing force under compression. Conversely, studies on biomimetic armour systems inspired by marine composites such as nacre from mollusc shells^4,22–26^, conch^20,27^ and fish scale^28,29^ are more dominant for protective applications due to their excellent damage tolerance.

Engineered cellular structures have been investigated thoroughly in the literature for numerous applications, including blast^3,6,30^, impact^3,31–33^ and crash-worthiness^5,34–36^. Examples include auxetic topologies with innovative geometries such as traditional re-entrant^3,6,31,37^, re-entrant with sinusoidal ribs^38^, star-shaped honeycombs^33^, double arrowhead^32^, spiral^5,36^, hexagonal^5,35,36^, and hybrids of re-entrant and rhombic honeycombs^39^. Although these types of structures have been shown to possess energy absorption characteristics, bio-inspired structures offer more complexity and design freedom, which can lead to futuristic protective technologies. Based on evidence from our previous study, where compression tests were conducted on several 3D printed designs of cellular structures^16^, bone-like scaffolds were shown to possess superior mechanical characteristics over their traditional honeycomb and re-entrant counterparts, which have been studied extensively in the literature^31,35,37^. Compared to the hexagonal structure, the bone-like structure showed a significant increase in energy absorption^16^. This prominent performance enhancement was facilitated by progressive buckling and collapse mechanisms, which were activated by the hybrid bone-like cells.

In this work, an analytical model is developed to predict the Young’s modulus, Euler buckling load and strain energy density of the 3D printed bone-like cellular structure. Exhaustive analytical simulations are then conducted in parallel to evolve the most effective cellular structure, whereby the energy absorption is used as the key performance criterion. The novel aspects of this study are highlighted by the development of a framework that can analytically capture the mechanical properties of hybrid bioinspired unit cell structures based on the intricate structural features of bone. Given that the design parameters can capture billions of unique unit cell topologies, including those with traditional honeycomb geometries such as hexagonal and re-entrant auxetics, the optimal unit cell can be extracted by covering all possibilities in a large space of design parameters.

## Analytical modelling and experimental methods

### Biomimicry to engineered cellular structure

**Figure 1:**
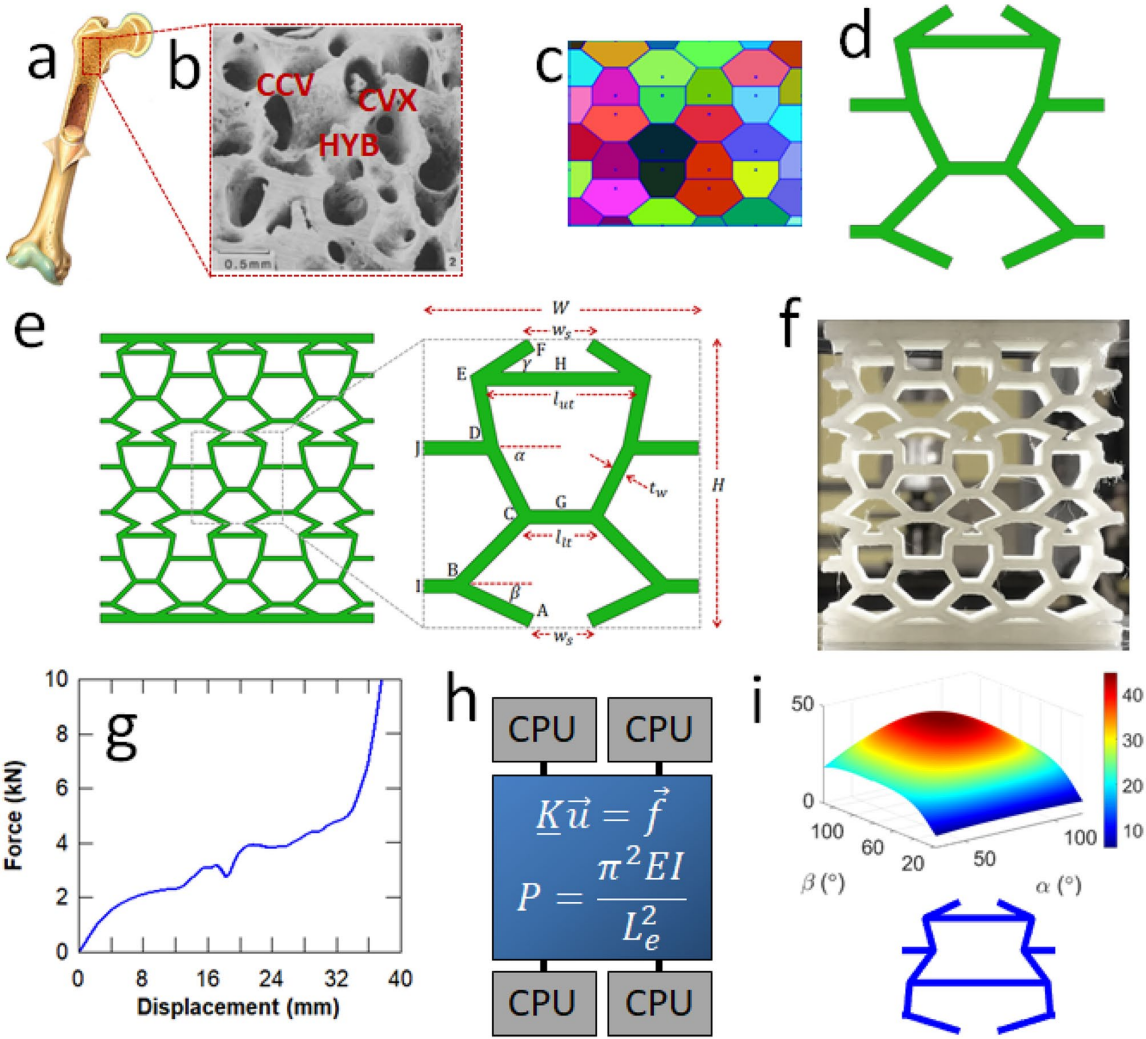
(**a**) Trabecular bone^16^; (**b**) Closed cell plate-likestructure of trabecular bone, which is composed of concave (CCV), convex (CVX) and hybrid (HYB), both concave and convex cells^16^; (**c**) Voronoi diagram for mimicking trabecular bone; (**d**) Unit cell extracted from the Voronoi diagram; (**e**) Bone-like structure generated from the unit cell; (**f**) 3D printed bone-like scaffold; (**g**) Force–displacement curve of a 3D printed bone-like structure; h) Parallel execution of structural analysis equations to obtain the optimal bone-like cellular structure; (**i**) Optimised bone-like unit cell.

Trabecular bone may possess a closed-cell plate-like structure, an open-cell strut-like structure or a hybrid of the two. Plate-like structures (Fig. 1a,b) can be found in denser bones that can support high loading demands, such as the human femur. Hybrid plate-like and strut-like structures typically exist at the interface between the compact (cortical) shell and the cellular (trabecular) core of bone. Trabecular bone typically has a relative density less than 70%^17^. More specifically, the relative density of open cell rod-like and closed cell plate-like trabecular structures is typically less than 0.13 and greater than 0.2, respectively. At intermediate relative densities, trabecular bone is a hybrid of rod- and plate-like elements^17^. Concave (CCV) and convex (CVX) cell geometries can be observed in the hybrid (HYB) plate-like cellular structure of trabecular bone (Fig. 1b). Mimicking these novel characteristics of trabecular bone can lead to the development of engineered cellular structures with superior energy absorption capabilities, by controlling their buckling and collapse mechanisms.

An overall snapshot of the analytical and experimental framework developed in this work is shown in Fig. 1, from the biomimetic conceptual level to the most optimal engineered cellular structure. The bone-like cellular structure (Fig. 1b) is mimicked using a periodic Voronoi diagram, which is an organised set of points (sites) that control the shape of its polygons (Fig. 1c)^40^. A unit cell (Fig. 1d) is extracted from the Voronoi diagram and manipulated to yield a bone-like CAD model with defined design parameters (Fig. 1e). The 3D printed bioinspired prototype (Fig. 1f) is generated from this CAD model and tested under compression. The analytical model is developed to predict the collapse load and Young’s modulus of the bone-like structure, and validated using the experimental force–displacement curve (Fig. 1g,h). Extensive simulations are executed in parallel to exhaustively manipulate the design parameters and identify the best bone-like unit cell that exhibits the highest energy absorption capacity (Fig. 1h,i).

### Design of bone-like unit cell

**Figure 2:**
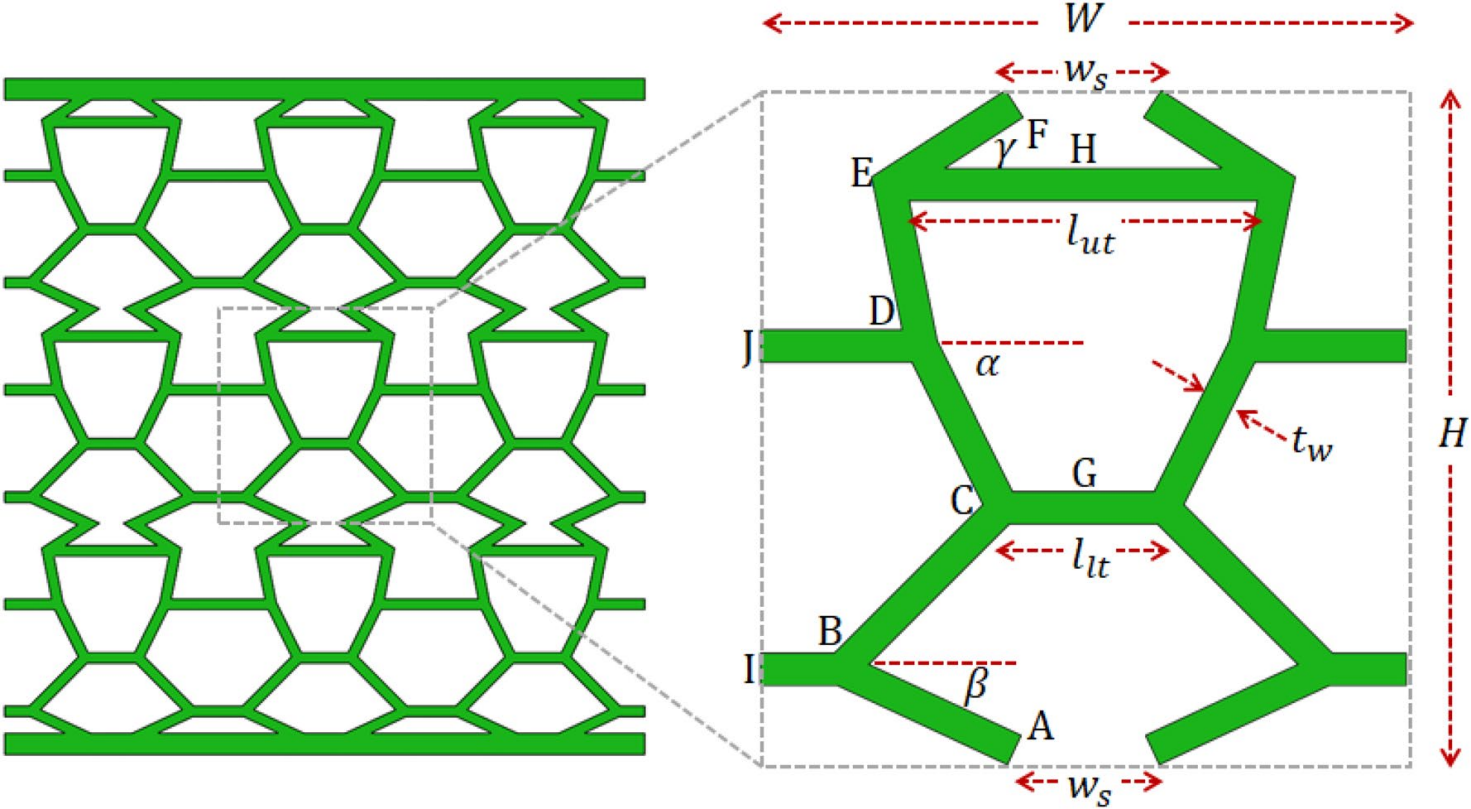
Bio-inspired structure and unit cell with the design parameters and nodes A–J labelled.

Conventional cellular structures, including re-entrant and honeycomb, are typically built from a single periodic unit cell^30,41^, which limits its design parameters. In contrast, the bioinspired unit cell (Fig. 2) is constructed with an upper and lower sub-cell, which provides more freedom in design. Comprehensive details on generating the 3D printed bioinspired unit cell using Voronoi diagrams and its demonstrated advantages over traditional hexagonal and re-entrant structures are provided in^15^. The design parameters of the bio-inspired unit cell are summarised as follows: H and W are the height and width of the unit cell, respectively; $\alpha$ and $\beta$ are the angles of the upper and lower sub-cell, respectively; $l_{ut}$ and $l_{lt}$ are the lengths of the upper and lower tie, respectively; $t_{w}$ is the thickness of the unit cell wall; and $t$ is the out-of-plane thickness of the unit cell. Note that the width of the unit cell $w_{s}$ can be determined from the above parameters, as demonstrated hereafter.

The lengths and angles of each element of the unit cell are tabulated below, noting that only half the cell will be analysed due to symmetry. The angles are determined with respect to the global horizontal axis.

Constraints are placed on the key design parameters to obtain a valid unit cell within the bounds $\left( {0,W} \right)$: $0 < w_{s} < W$; $0 < b < W$; $0 < l_{lt} < W$; $0 < g < W$; and $0 < l_{ut} < W$.

The relative density can be inferred from the lengths of the elements in Table 1 as follows:

1 $$\frac{\rho }{{\rho_{S} }} = \frac{{2t_{w} \sum L_{e} }}{WH}$$

where $\rho$ is the density of the cellular structure; $\rho_{S}$ is the density of the solid material; $t_{w}$ is the thickness of the cell walls; $L_{e}$ is the length of element e; and W and H are the width and height of the unit cell, respectively.

**Table 1:** Element lengths and angles of the bone-like half unit cell (see Fig. 2 for a visual).

Element	Length	Angle
AB	$L_{AB} = \frac{H}{8sin\beta }$	$\phi_{AB} = \pi - \beta$
BC	$L_{BC} = \sqrt {d^{2} + \left( \frac{H}{4} \right)^{2} }$	$\phi_{BC} = \tan^{ - 1} \left( \frac{H}{4d} \right)$
CD	$L_{CD} = \frac{H}{4sin\alpha }$	$\theta_{CD} = \pi - \alpha$
DE	$L_{DE} = \sqrt {f^{2} + \left( \frac{H}{4} \right)^{2} }$	$\phi_{DE} = \frac{\pi }{2} + \tan^{ - 1} \left[ \frac{4f}{H} \right]$
EF	$L_{EF} = \sqrt {e^{2} + \left( \frac{H}{8} \right)^{2} }$	$\phi_{EF} = \gamma$
EH	$L_{EH} = \frac{{l_{ut} }}{2}$	$\phi_{EH} = 0$
CG	$L_{CG} = \frac{{l_{lt} }}{2}$	$\phi_{CG} = 0$
JD	$L_{JD} = \frac{{W - l_{lt} - 2c}}{2}$	$\phi_{JD} = 0$
IB	$L_{IB} = \frac{W - b}{2}$	$\phi_{IB} = 0$

$$*a = \frac{H}{8tan\beta }; b = w_{s} + 2a; c = \frac{H}{4tan\alpha }; d = \frac{{b - l_{lt} }}{2}; e = \frac{H}{8tan\gamma }; f = \frac{{ l_{ut} - l_{lt} - 2c}}{2}; g = l_{lt} + 2c;w_{s} = l_{ut} - 2e$$.

### Analytical model development

The deformation of a cellular structure can be predicted by applying the direct stiffness method of structural analysis to the bio-inspired unit cell design shown in Fig. 2^42^. The forces and moments in each element are related to the displacements and rotations in the global coordinate system as follows:

2 $$\underline{T}_{e} \underline{K}_{e} \underline{T}_{e}^{T} \vec{u}_{Ge} = \vec{f}_{Ge}$$

where $\underline{T}_{e}$ is the transformation matrix from the local to global coordinate system; $\underline{K}_{e}$ is the stiffness matrix of element e, which accounts for axial and flexural deformations^42^; $\vec{u}_{Ge} = \left[ {u_{Gi}\;v_{Gi}\;\theta_{i}\;u_{Gj}\;v_{Gj}\;\theta_{j}} \right]^{T}$ is the element displacement vector in global coordinates; and $\vec{f}_{Ge} = \left[ {f_{Gi|x}\;f_{Gi|y}\;m_{i}\;f_{Gj|x}\;f_{Gj|y}\;m_{j} } \right]^{T}$ is the element force vector in global coordinates. Each element stiffness matrix $\underline{K}_{e}$ is expanded into the global stiffness matrix of the structure i.e. $\underline{K}_{G} = \sum \underline{K}_{e}^{exp}$.

**Figure 3:**
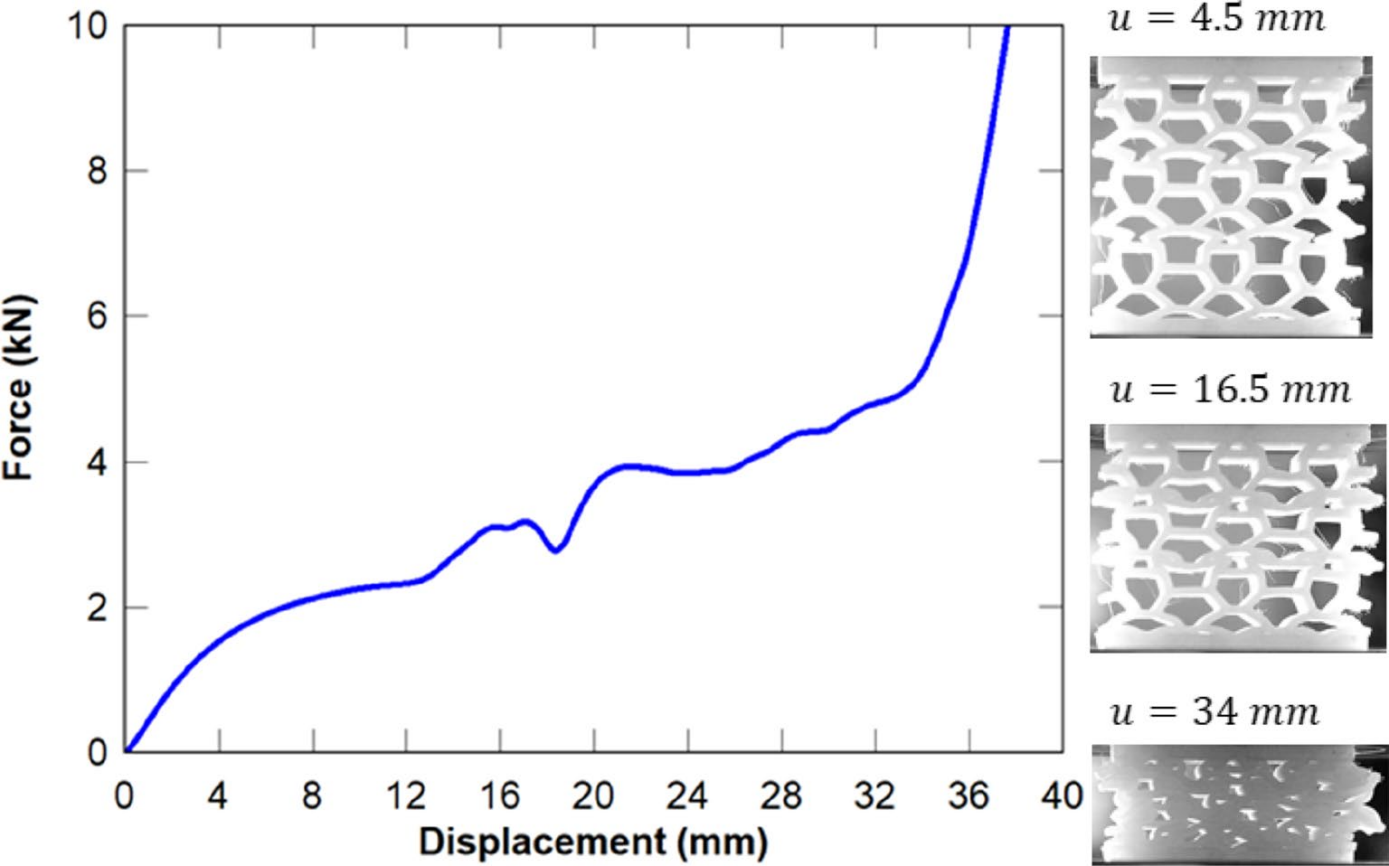
Compressive force–displacement curves for the 3D printed bone-like cellular structure^16^. The deformations in the linear-elastic, plastic and densification regions are also illustrated.

The boundary conditions are determined based on the symmetry of the unit cell: horizontal sway-rigid support at node A $\left( {v_{Ga} = \theta_{a} = 0} \right)$; and vertical sway-rigid support at nodes F to J $\left( {u_{G} = \theta = 0} \right)$. The stress, strain and Young’s modulus of the unit cell can be written as follows:

3 $$\sigma = \frac{P}{2nWt};\varepsilon = \frac{{v_{Gf} }}{H};E = \frac{PH}{{2nWtv_{Gf} }}$$

where the force $P$ is arbitrarily chosen in the linear-elastic region (Fig. 3), $n$ is the number of unit cells across the width of the structure, $W$ and $H$ are the width and height of a single unit cell, $t$ is the out-of-plane thickness of the unit cell, and $v_{Gf}$ is the global vertical displacement at node F.

Buckling of the unit cell is dictated by the critical column, where each column is supported by elastic restraints. The critical buckling load of an elastically supported column can be inferred by solving the Euler buckling ordinary differential equation of a pinned–pinned column and applying elastic boundary conditions to the pinned joints^43,44^. This well-known equation and its solution are represented as follows:

4a $$v^{{\prime \prime }} + \lambda^{2} v = 0$$

4b $$v\left( x \right) = A \hbox{cos} \lambda x + B \hbox{sin} \lambda x$$

where $v$ is the lateral displacement, $\lambda^{2} = P{/}E_{S} I$ and $E_{S} I$ is the flexural rigidity of the column; P is the compressive load; and $A$ and $B$ are constants of integration. By differentiating Eq. (4) twice and imposing elastic boundary conditions at the joints of the column (at $x = 0 \;{\text{and}}\;x = L$), the following expressions are obtained:

5a $$v\left( 0 \right) = 0 \to A = v\left( 0 \right)$$

5b $$v\left( L \right) = A\hbox{cos} \lambda L + B\hbox{sin} \lambda L$$

5c $$v^{{\prime }} \left( 0 \right) = - \lambda B \to B = - \frac{{v^{{\prime }} \left( 0 \right)}}{\lambda }$$

5d $$v^{{\prime }} \left( L \right) = - \lambda A\hbox{sin} \lambda L - \lambda B\hbox{cos} \lambda L$$

5e $$v^{{\prime \prime }} \left( 0 \right) = - \lambda^{2} A = \frac{M\left( 0 \right)}{{E_{S} I}} \to A = - \frac{1}{{\lambda^{2} }}\frac{M\left( 0 \right)}{{E_{S} I}}$$

5f $$v^{{\prime \prime }} \left( L \right) = - \lambda^{2} A\hbox{cos} \lambda L + \lambda^{2} B\hbox{sin} \lambda L = \frac{M\left( L \right)}{{E_{S} I}}$$

where $M\left( 0 \right)$ and $M\left( L \right)$ are the moments at the joints of the column, and $A$ and $B$ are constants. By combining Eqs. (5a–f), the following expression can be obtained, which relates the rotations at the joints of the column with the bending moment at the joint at $x = L$:

6 $$\frac{M\left( L \right)}{{E_{S} I}}\frac{1}{{\lambda^{2} \hbox{cos} \lambda L}} - \frac{{v^{{\prime }} \left( L \right)}}{\lambda \hbox{sin} \lambda L} = - \frac{{v^{{\prime }} \left( 0 \right)}}{\lambda }\frac{\hbox{sin} \lambda L}{{\hbox{cos} \lambda L}} - \frac{{v^{{\prime }} \left( 0 \right)\hbox{cos} \lambda L}}{\lambda \hbox{sin} \lambda L}$$

The rotation at the joint at $x = L$ can be written in terms of the bending moment $M\left( L \right)$ and the stiffness of the restraining member:

7 $$v^{{\prime }} \left( L \right) = - \frac{M\left( L \right)}{{K_{L} }}$$

where $K_{L}$ is the stiffness of the joint at $x = L$. The negative sign signifies that the bending moment in the bracing element opposes the bending moment in the column. Substituting Eq. (7) into Eq. (6) gives the following relationship:

8 $$M\left( L \right) = - \frac{{v^{{\prime }} \left( 0 \right)\left( {\hbox{sin}\lambda L{\text{tan}} \lambda L + \hbox{cos} \lambda L} \right)}}{{\frac{1}{{E_{S} I}}\frac{\tan \lambda L}{\lambda } + \frac{1}{{K_{L} }}}}$$

where $E_{S} I$ is the bending stiffness of the solid material; $\lambda = \sqrt {P/E_{S} I}$ and $P$ is the compressive force in the column; $v^{{\prime }} \left( 0 \right)$ is the rotation at $x = 0$; $L$ is the length of the column; and $K_{L} = - M\left( L \right){/}v^{{\prime }} \left( L \right)$ is the stiffness of the joint. By inspection, buckling occurs when small changes in the bending moment $M\left( L \right)$ produce significant changes in the rotation^43^. This condition is reached when the denominator of Eq. (8) approaches zero, which yields the following nonlinear solution for $\lambda$ and the critical buckling load $P_{CR}$:

9 $$\frac{{\text{tan}} \lambda L}{{\lambda L}} = - \frac{{L_{t} }}{\alpha L}; \quad P_{CR} = \lambda^{2} E_{S} I$$

where $\alpha$ is the stiffness coefficient of the joint; $L_{t}$ is the length of the restraining tie; and $L$ is the length of the column element being analysed for buckling. The smallest root $\lambda$ for Eq. (9) can be obtained graphically for each combination of $L_{t} {/}L$ and recorded in a design table.

The energy absorbed by the half unit cell can then be calculated from Eqs. (3) and (9) as follows, assuming elastic buckling:

10 $$U = \frac{{\sigma_{CR}^{2} }}{2E}$$

where $\sigma_{CR}$ is the critical buckling stress $(P_{CR} {/}A)$ and $A$ is the cross-sectional area of the column element. The mass of the unit cell can be calculated as follows:

11 $$m = \rho_{S} t_{w} t\mathop \sum \limits_{i} L_{i}$$

where $\rho_{S}$ is the density of Markforged Nylon, namely $1.1\,{\text{kg/m}}^{3}$, $t_{w}$ is the thickness of the cell walls, $t$ is the out-of-plane thickness of the unit cell and $L_{i}$ is the length of each element.

## Results and discussion

### Experimental validation

The analytical model is validated using the results from our compressive tests on the bone-like cellular structure, which are presented in Fig. 3^16^. The goal is to verify that buckling is the primary collapse mechanism of the structure as opposed to plastic deformation in the material. The following parameters are used for validating the analytical model, which are identical to the tested cellular structure (see Fig. 3): $W = H = 20\,{\text{mm}}$; $\alpha = 63.43^\circ , \beta = 25^\circ$, $\gamma = 41.14^\circ$; $l_{ut} = 10\,{\text{mm}}$, $l_{lt} = 5\,{\text{mm}}$; $t_{w} = 2\,{\text{mm}}$; $t = 30\,{\text{mm}}$; $E_{S} = 320\,{\text{MPa}}$ (Markforged Tough Nylon, 3D printing material). A force of 1,000 N is chosen arbitrarily within the linear elastic region of the force–displacement curve (Fig. 3), and divided over the number of half unit cells (six); the force per half unit cell is thereby $166.67\,{\text{N}}$. A vertical displacement of $v_{Gf} = 0.84\,{\text{mm}}$ at node F $\left( {v_{Gf} } \right)$ was predicted from the analytical model by solving Eq. (2). The vertical displacement at node F $v_{Gf}$ is used to calculate the strain and Young’s modulus from Eq. (3). The critical buckling load was then calculated using Eq. (9) for each column and presented in Table 3.

It can be inferred from Table 2 that the predicted Young’s modulus of the half unit cell is in agreement with the experiment within 7%. The experimental critical buckling load is also within the range of the predicted results. Although column AB provided the closest buckling load (Table 3) with the experiment, it is difficult to predict that this column will first buckle, as the condition of instability depends on imperfections that are inherent in the 3D printing process, as well as the load transfer through the structure. Regardless, the analytically predicted Young’s modulus and critical buckling load are conservative for design purposes. Importantly, the predicted minimum critical buckling stress (in column BC) is well within the linear elastic region of the material curve of the Markforged Tough Nylon 3D printing material^16^, which verifies that buckling is the primary collapse mechanism of the structure as opposed to plastic collapse. The effective lengths and $k$ factors are calculated from the Euler buckling load and presented in Table 3, which are slightly larger than 1 as expected, noting that $k = 1$ represents a sway rigid-to-rigid column with one point of inflexion or a braced pinned-to-pinned column. The critical buckling loads listed in Table 3 signify that buckling can occur in either column AB, BC, CD or DE. Column EF is ignored because the critical buckling stress exceeds the linear elastic limit of the material (see^16^). Hence, for the optimisation studies conducted hereafter, the critical buckling load is calculated for columns AB to EF for each unit cell configuration and the minimum buckling load is used to compute the strain energy density.

**Table 2:** Experimental validation of the analytical model.

	Analytical	Experimental^16^
Displacement $\left( {v_{Gf} } \right)$	$0.84\;{\text{mm}}$ (half unit cell)	$2.63 \;{\text{mm}}$ (structure)
Young’s modulus $\left( E \right)$	13.2 MPa	14.1 MPa
Critical buckling load $\left( {P_{CR} } \right)$	930–1,340.7 N	1,220 N
Critical buckling stress $\left( {\sigma_{CR} } \right)$	15.5–22.3 MPa	20.3 MPa

**Table 3:** Euler buckling load for each column of the analysed half unit cell (Fig. 2).

Column	Critical buckling load ($P_{CR}$ N)	Effective length ($L_{e}$ mm)	k factor ($L_{e} /L$)
AB	1,267.4	7.06	1.194
BC	930	8.24	1.166
CD	1,115.6	7.52	1.346
DE	1,340.7	6.86	1.346
EF	2,105 (N/A)	N/A	N/A

### Optimal design parameters

A computer algorithm was developed to execute many parallel simulations to obtain the design parameters ($\alpha ,\beta ,\gamma ,l_{ut} ,l_{lt}$) that produce the biomimetic unit cell with the optimal energy absorption. To this effect, the sub-cell angles and tie lengths were modified as follows: $\alpha ,\beta ,\gamma{:} \, \left[ {5,175^\circ } \right]$ in increments of $1^\circ$; and $l_{ut} ,l_{lt}{:} \left[ {1,19\,{\text{mm}}} \right]$ in increments of 1 mm. This equates to 1.7 billion cases, which were run on a supercomputer using 170 threads (total runtime of 2.3 h). The results are reported in Table 4 and analysed herein. Case 1 ($\alpha = \beta = \gamma = 90^\circ , l_{ut} = l_{lt} = 1\,{\text{mm}}$) results in vertical columns and produces the stiffest structure with the lowest energy absorption. This is expected because the columns are more significantly rigid under axial deformation as opposed to flexure. Case 2 ($\alpha = 79^\circ , \beta = 30^\circ , \gamma = 34^\circ , l_{ut} = l_{lt} = 9\,{\text{mm}}$) results in a structure with a significantly low stiffness and a high buckling load compared to Case 1. This effectively increases the energy absorption relative to Case 1 according to Eq. (10). The design parameters are analysed individually in the following sub-section to gain further insight into this behaviour. Case 3 ($\alpha = 122^\circ , \beta = 20^\circ ,\gamma = 29^\circ , l_{ut} = 14\,{\text{mm}}, l_{lt} = 18\,{\text{mm}}$) produces the most optimal structure with the lowest stiffness and highest energy absorption. Although the Euler buckling load is nearly identical to Case 1, the stiffness is significantly lower. According to Eq. (9), the buckling load depends on the ratio of the length of the tie restraint $L_{t}$ to the length of the restrained column. The mechanical properties of the re-entrant auxetic structure, which is extensively investigated in the literature for protective applications, are also presented in Table 4 for comparison. It is evident that the bioinspired structure shows a significantly higher energy absorption capacity with a slight increase in mass (around 4%) under quasi-static loading. This promising result can motivate further numerical investigations to obtain the optimal bioinspired structure under extreme loadings in future work. The design parameters are also analysed individually in the following sub-section to gain further insight into this behaviour.

**Table 4:** Optimal unit cells obtained by modifying all five design parameters simultaneously, namely the cell wall angles and tie lengths. The re-entrant auxetic topology is also presented as a benchmark case.

Maximum specific properties (MPa/g)	Other specific properties (MPa/g)	Angles	Tie lengths (mm)	Unit cell
$E_{max} = 24.2$	$\sigma_{CR} = 6.8 \left( {CD} \right)$ $U = 0.94$	$\alpha = 90^\circ$ $\beta = 90^\circ$ $\gamma = 90^\circ$	$l_{ut} = 1$ $l_{lt} = 1$	$m = 2.64\;{\text{g}}$
$\sigma_{CR|max} = 7.9 \left( {CD} \right)$	$E = 6.7$ $U = 4.6$	$\alpha = 79^\circ$ $\beta = 30^\circ$ $\gamma = 34^\circ$	$l_{ut} = 9$ $l_{lt} = 9$	$m = 2.84\;{\text{g}}$
$U_{max} = 5.8$	$E = 2.7$ $\sigma_{CR} = 5.6 \left( {AB} \right)$	$\alpha = 122^\circ$ $\beta = 20^\circ$ $\gamma = 29^\circ$	$l_{ut} = 14$ $l_{lt} = 18$	$m = 3.25\;{\text{g}}$
$U_{max} = 1.5$	$E = 10.1$ $\sigma_{CR} = 5.5$	$\alpha = 116.6^\circ$ $\beta = 116.6^\circ$ $\gamma = 63.43^\circ$	$l_{ut} = 12.5$ $l_{lt} = 12.5$	$m = 3.13\;{\text{g}}$

### Influence of individual design parameters

**Figure 4:**
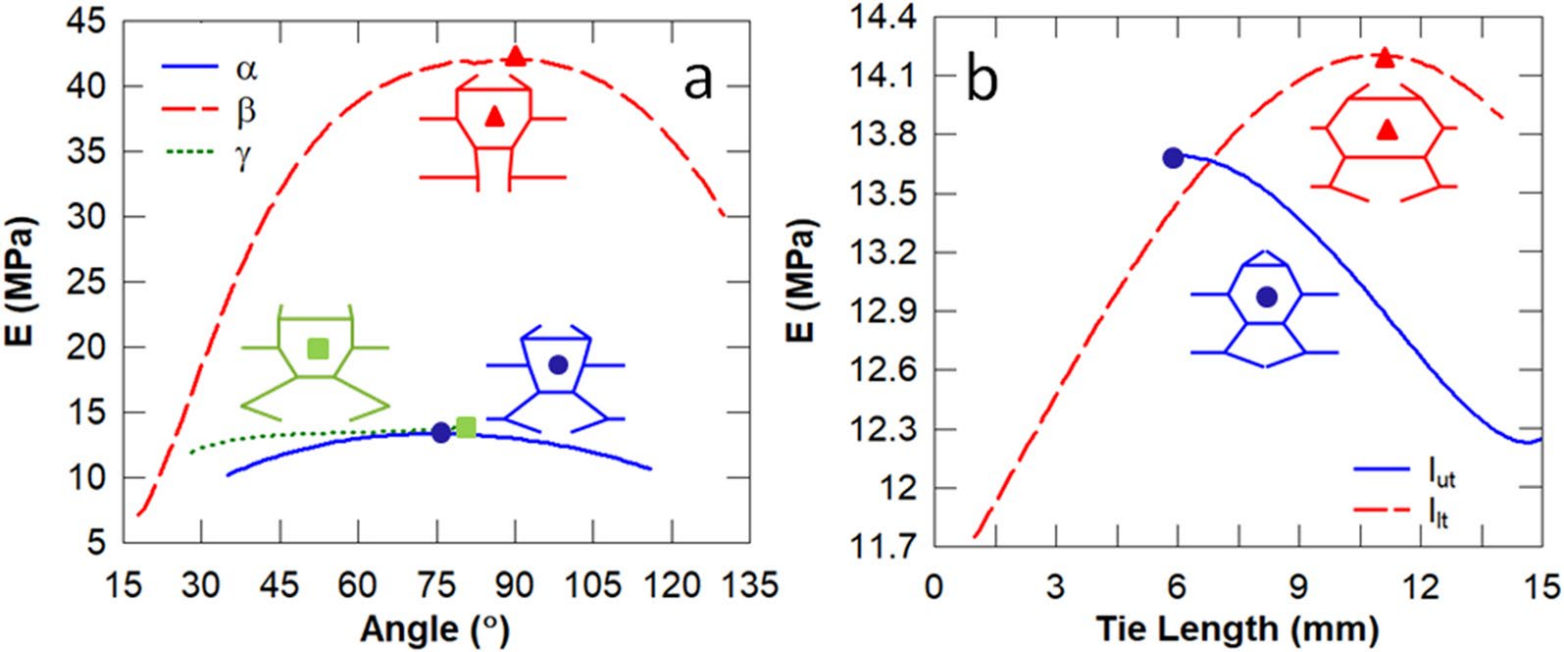
Influence of the sub-cell angles (**a**) and tie lengths (**b**) on the stiffness. The unit cell corresponding to the peak stiffness is also illustrated.

In this set of studies, the influence of each individual design parameter of the biomimetic unit cell (see Fig. 2) on its energy absorption, stiffness and Euler buckling stress is analysed. To this effect, the parameters of the baseline unit cell are modified individually from the reference baseline configuration ($\alpha = 63.43^\circ , \beta = 25^\circ$, $\gamma = 41.14^\circ$, $l_{ut} = 10\,{\text{mm}}$ and $l_{lt} = 5\,{\text{mm}}$). It is evident in Fig. 4 that the sub-cell angle $\beta$ has the most significant influence on the stiffness of the unit cell, followed by the length of the lower tie $l_{lt}$. It is expected that $\beta = 90^\circ$ results in the stiffest unit cell because the lower sub-cell columns become parallel such that they transmit the load via axial deformation predominantly, as opposed to flexure.

**Figure 5:**
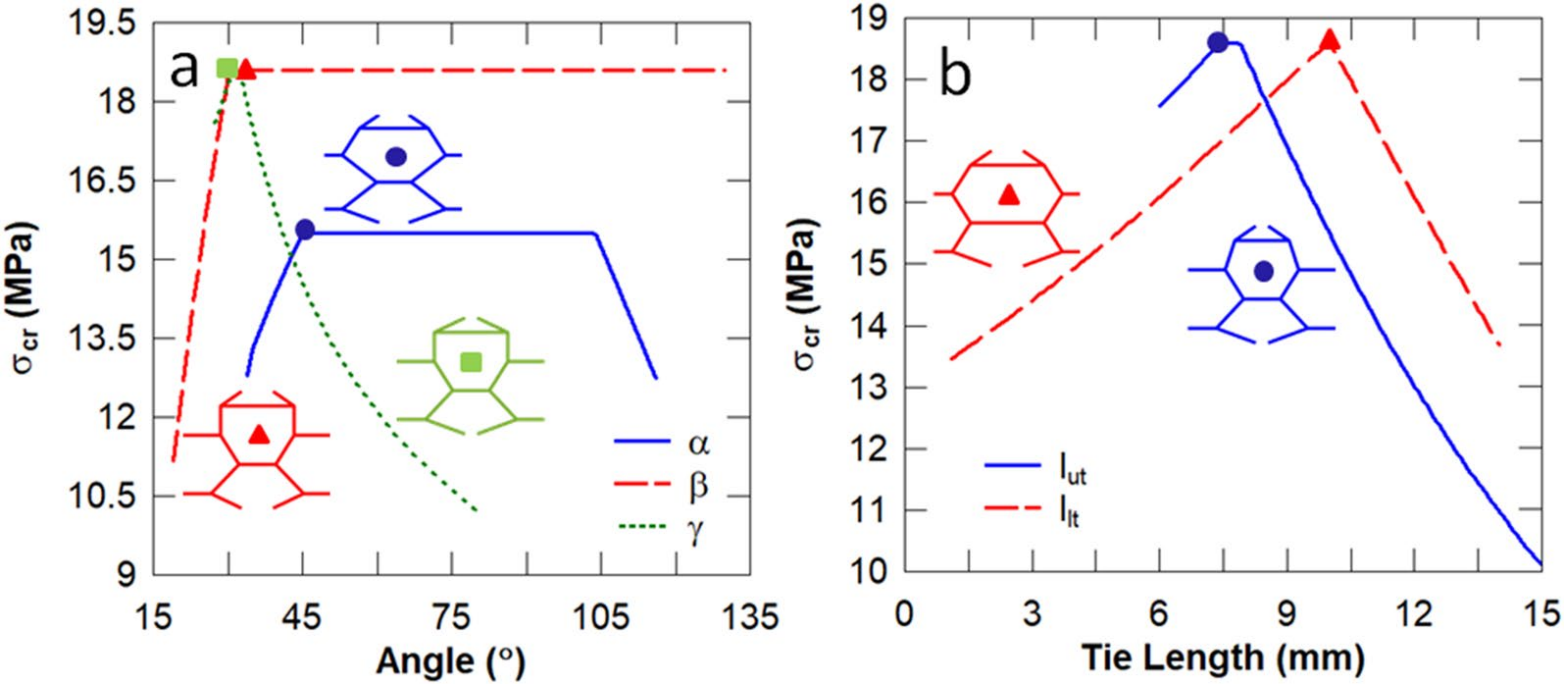
Influence of the sub-cell angles (**a**) and tie lengths (**b**) on the Euler buckling stress. The unit cell corresponding to the peak Euler buckling stress is also illustrated.

It can be observed in Fig. 5 that both sub-cell angles $\beta$ and $\gamma$, and the tie lengths $l_{ut}$ and $l_{lt}$ produce the unit cell with the highest Euler buckling stress. According to Eq. (9), the Euler buckling load is governed by the ratio of the length of the tie restraint ($L_{t}$) to the length of the restrained column ($L$). The ratio $L_{t} {/}L$ and the critical column are listed in Table 5 for each individual design parameter that produced the maximum buckling stress. It can be deduced that the plateau region e.g. for $45^\circ < \alpha < 103^\circ$ gives the same $L_{t} {/}L$ ratio and thereby produces an identical Euler buckling stress.

**Table 5:** Maximum buckling stress obtained for each design parameter, and its corresponding critical column and $L_{t} /L$ ratio. The reader may refer to Fig. 2 to identify the critical columns.

Design parameter	Critical column	$L_{t}$ (mm)	$L$ (mm)	$L_{t} /L$	$\sigma_{CR}$ (MPa)
$\alpha = 45^\circ$	BC	2.5	7.07	0.35	15.5
$\beta = 31^\circ$	CD	5	5.59	0.89	18.6
$\gamma = 31^\circ$	CD	5	5.59	0.89	18.6
$l_{ut} = 7.4 \;{\text{mm}}$	CD	5	5.59	0.89	18.6
$l_{lt} = 10 \;{\text{mm}}$	BC	5	5.59	0.89	18.6

**Figure 6:**
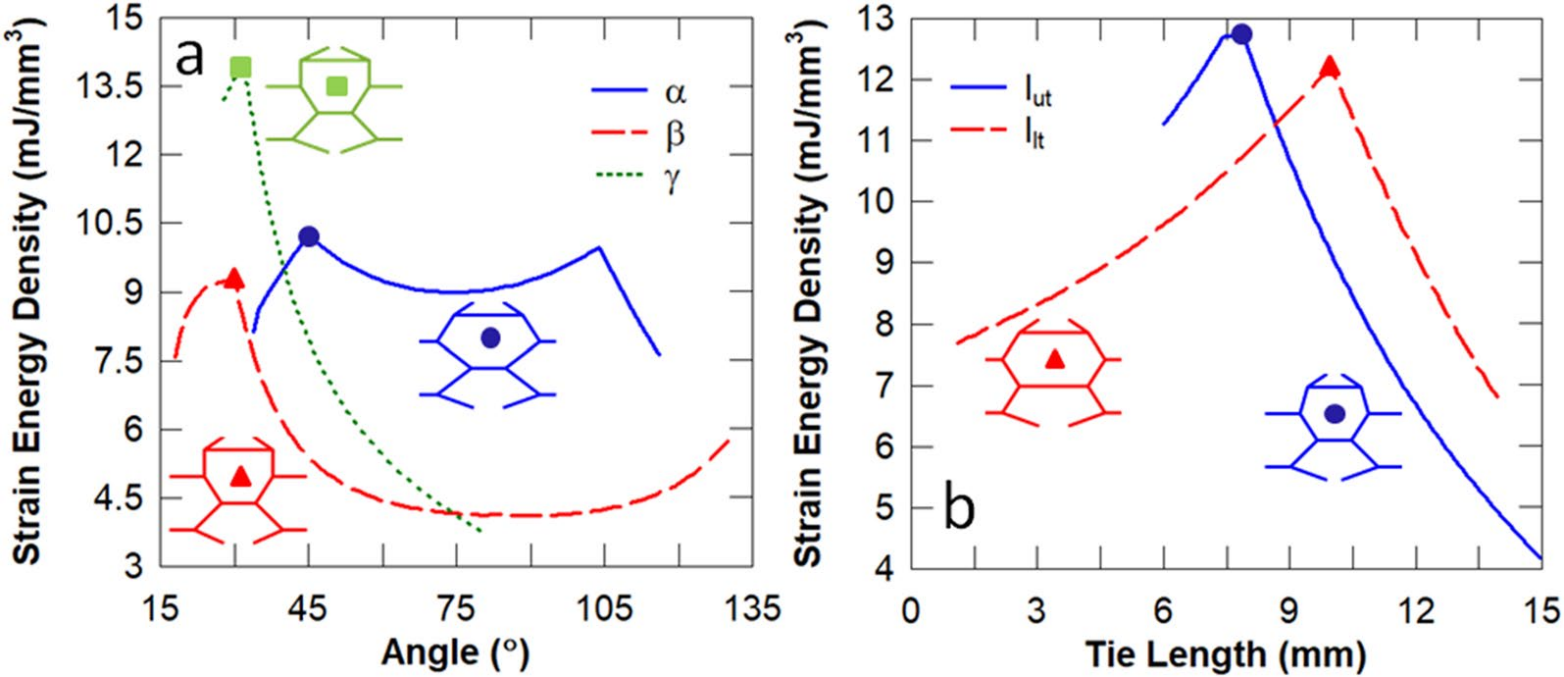
Influence of the sub-cell angles (**a**) and tie lengths (**b**) on the energy absorption. The unit cell corresponding to the peak strain energy density is also illustrated.

It is evident in Fig. 6 that the sub-cell angle $$\gamma =$$$$31^\circ$$ and upper cell wall angle of $$l_{ut} = 7.8\,{\text{mm}}$$ result in unit cells with the highest strain energy density. According to Eq. (10), the optimal energy absorption can be obtained by minimising the Young’s modulus E whilst maximising the critical buckling stress $$\sigma_{CR}$$. According to the five-parameter optimisation in Table 4, modifying each design parameter individually will significantly underestimate the unit cell with the maximum strain energy density ($$U_{max} = 18.8 \,{\text{mJ/mm}}^{3}$$). It can be deduced from Figs. 4 and 5 that minimising the sub-cell angle $$\gamma$$ produces a relatively constant low stiffness $$\sim 13\,{\text{MPa}}$$ and a maximum buckling stress of $$\sim 19\,{\text{MPa}}$$, respectively. The sub-cell angle $$\beta$$ and lower tie length $$l_{lt}$$ can overcome this restriction to reduce the stiffness further and thereby obtain the unit cell with the optimal energy absorption (see Fig. 4).

### Influence of combined design parameters

**Figure 7:**
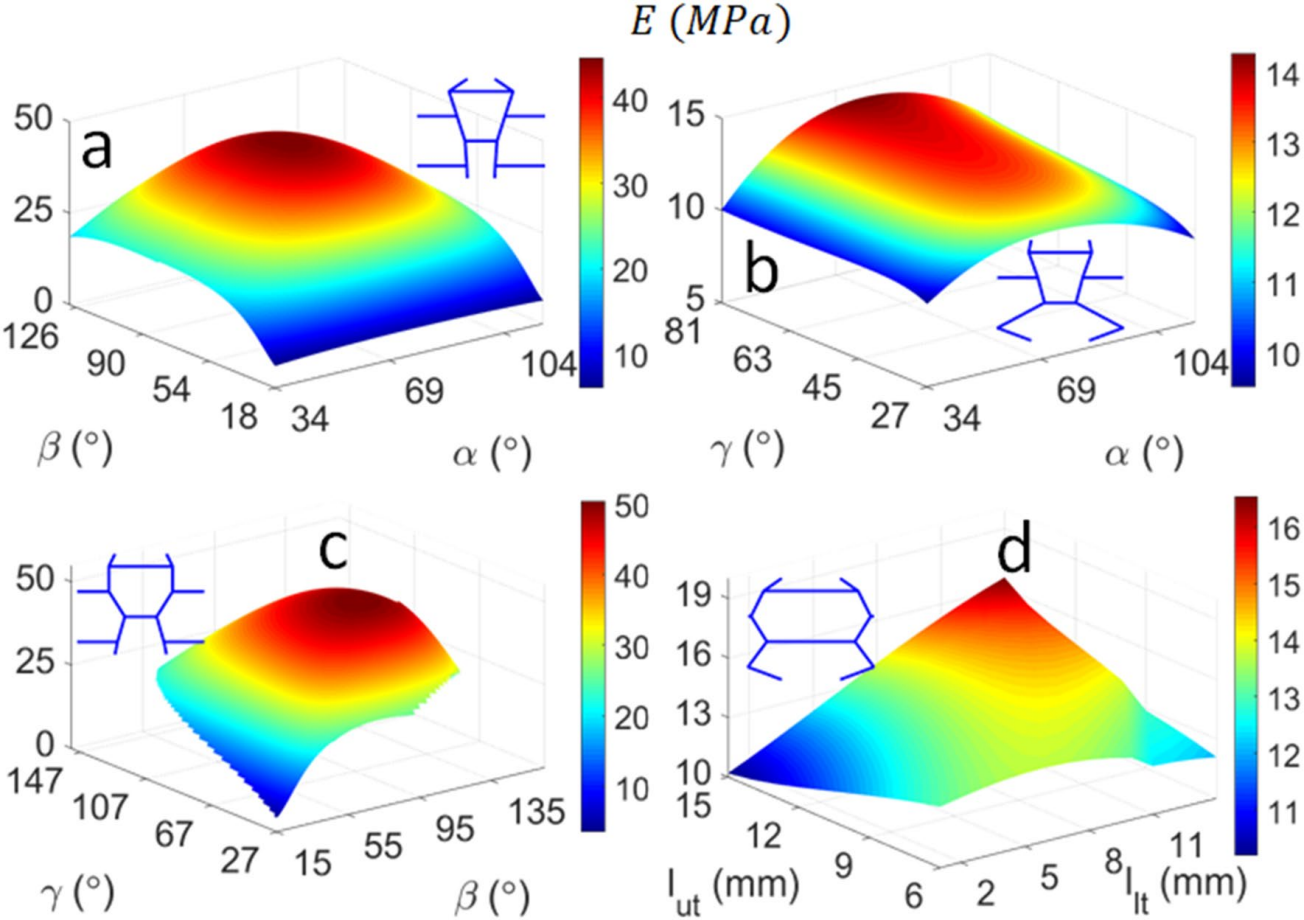
Influence of combined design parameters on the stiffness of the biomimetic unit cell: (**a**) $\alpha ,\beta$; (**b**) $\alpha ,\gamma$; (**c**) $\beta ,\gamma$; and (**d**) $l_{ut} , l_{lt}$. The unit cell corresponding to the peak stiffness is also illustrated.

In this set of studies, the combined effects of two design parameters of the biomimetic unit cell (see Fig. 2) on its energy absorption, stiffness and Euler buckling stress are analysed. The parameters of the baseline unit cell are modified from the reference configuration $\alpha = 63.43^\circ ,{ }\beta = 25^\circ$, $\gamma = 41.14^\circ$; $l_{ut} = 10\,{\text{mm}}$, $l_{lt} = 5\,{\text{mm}}$. The corresponding unit cells with the maximum values are also illustrated near each surface plot. It is evident from Fig. 7a,c that the sub-cell angle ${\upbeta }$ (see Fig. 2) is a key design parameter that dictates stiffness, whereby the lower columns of the unit cell are relatively vertical. This trend is consistent with that observed in Fig. 4, which showed that modifying $\beta$ alone produces the stiffest unit cell by far. The unit cell with the highest stiffness ($E_{max} = 50.3\,{\text{MPa}}$) is obtained when $\beta = 99^\circ$ and $\gamma = 69^\circ$ (Fig. 7c). This is an expected result, given that the columns of both sub-cells are relatively vertical, which promotes axial deformation over flexure. A relatively stiff unit cell ($E = 44.7\,{\text{MPa}}$) can also be obtained by other combinations of sub-cell angles e.g. $\alpha = 76^\circ$ and $\beta = 88^\circ$ (Fig. 7a), which further reinforces that the stiffness is controlled by $\beta$ as a governing parameter. In contrast, Fig. 7b,d show that the unit cells with the highest stiffness have more oblique members such that flexural deformation becomes more prominent, which consequently reduces the stiffness of the structure.

**Figure 8:**
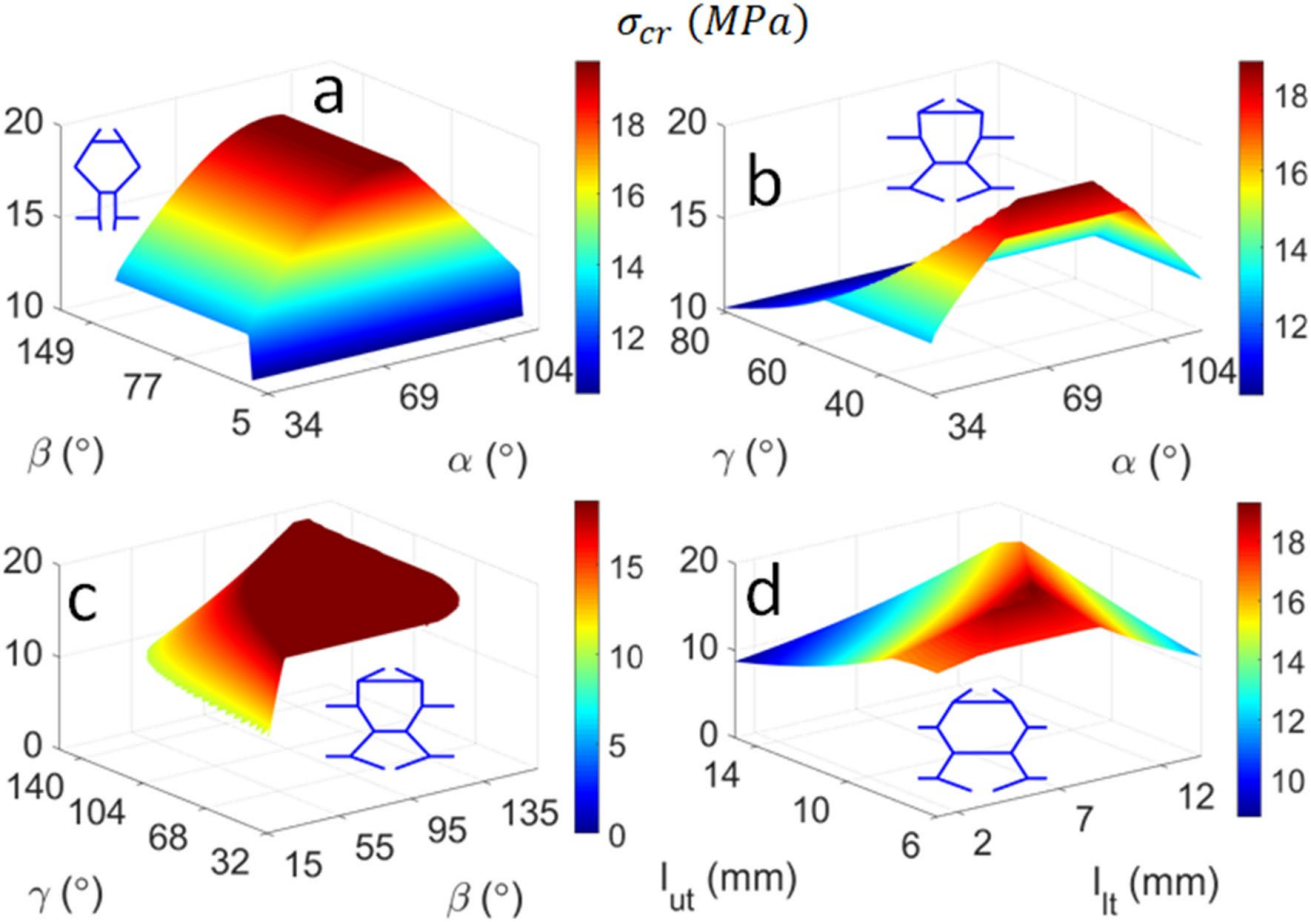
Influence of combined design parameters on the Euler buckling stress of the biomimetic unit cell: (**a**) $\alpha ,\beta$; (**b**) $\alpha ,\gamma$; (**c**) $\beta ,\gamma$; and (**d**) $l_{ut} , l_{lt}$. The unit cell corresponding to the peak buckling stress is also illustrated.

The highest critical buckling stress (${\upsigma }_{{{\text{CR}}}} = 19.7\,{\text{MPa}}$) is obtained from ${\upalpha } = 80^\circ$ and ${\upbeta } = 33^\circ$ (Fig. 8a). This is followed by ${\upsigma }_{{{\text{CR}}}} = 19.3\,{\text{MPa}}$, which results from the tie lengths ${\text{l}}_{{{\text{ut}}}} = {\text{l}}_{{{\text{lt}}}} = 9\,{\text{mm}}$ (Fig. 8d). From the discussion preceding Fig. 5 and Table 5, and referring to Eq. (9), the critical buckling load depends on the ratio of the restraining tie length to the length of the restrained column ${\text{L}}_{{\text{t}}} {\text{/L}}$. It is evident that the unit cell in Fig. 8a has the shortest tie (attached to longer columns CD and DE), which effectively yields a small ${\text{L}}_{{\text{t}}} {\text{/L}}$ ratio and thereby attracts a higher buckling stress. Longer plateau regions can be observed in the plots of Fig. 8b,c because the unit cells that produce the highest buckling stress have a more uniform structure, whereby the relative lengths between the struts and ties are similar.

**Figure 9:**
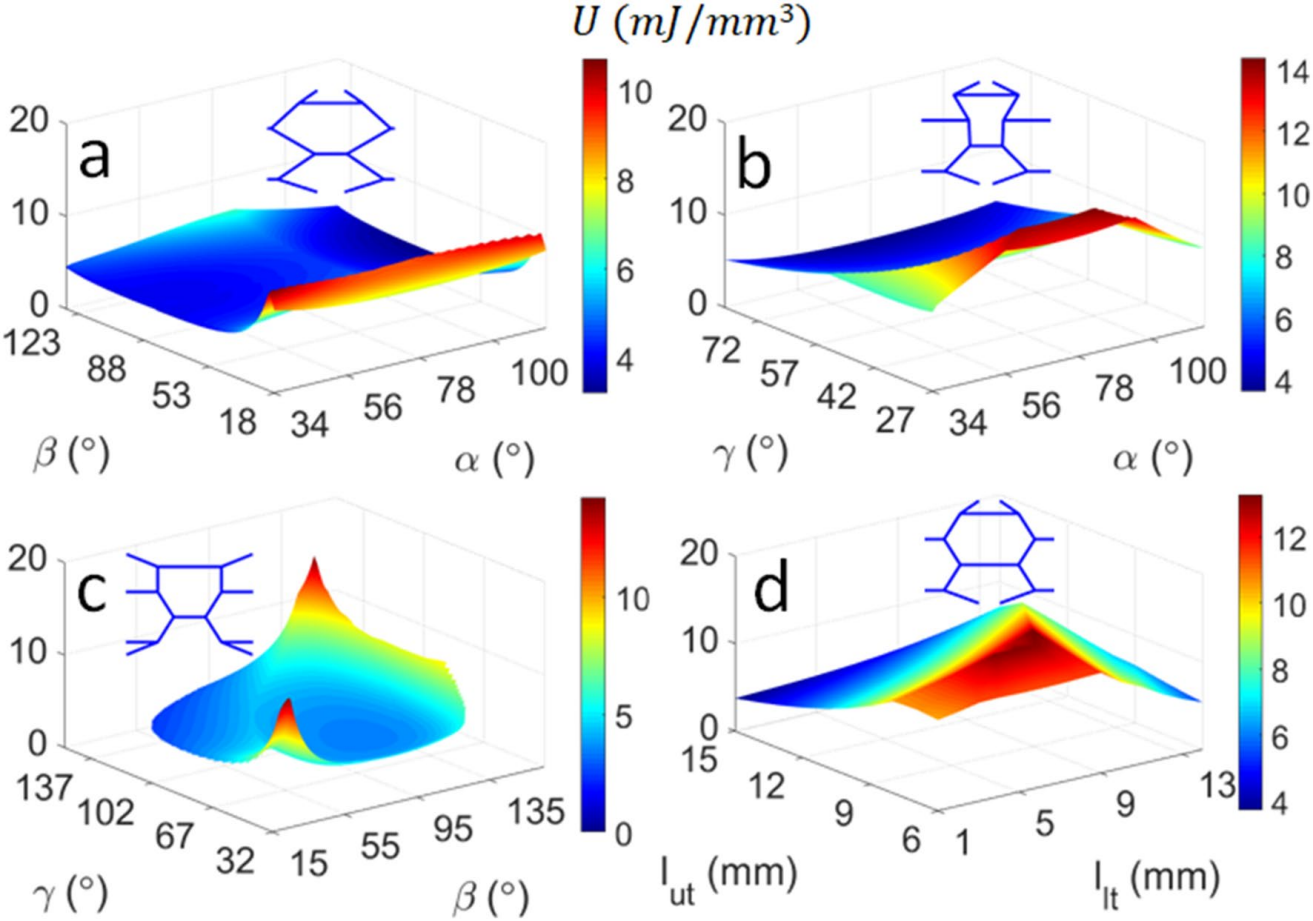
Influence of combined design parameters on the energy absorption of the biomimetic unit cell: (**a**) $\alpha ,\beta$; (**b**) $\alpha ,\gamma$; (**c**) $\beta ,\gamma$; and (**d**) $l_{ut} , l_{lt}$. The unit cell corresponding to the peak strain energy density (SED) is also illustrated.

The unit cell with the highest strain energy density (${\text{U}}_{{\max}} = 14.4\,{\text{MPa}}$) is obtained when ${\upalpha } = 88^\circ$ and ${\upgamma } = 32^\circ$ (Fig. 9b). Recalling from Eq. (10) and the discussion preceding Fig. 6 that the peak strain energy density is obtained when the critical buckling stress is maximised and the stiffness is minimised, it is evident that the unit cell illustrated Fig. 9b is designed to balance axial and flexural deformation, whereby the oblique orientations of its columns reduce the stiffness. The unit cell also has relatively similar column and tie lengths, which thereby produces a relatively high critical buckling load ($\sigma_{CR} = 18.9\,{\text{MPa}}$). Note that the critical buckling load does not vary within a large interval compared to the stiffness, given that the $L_{t} {/}L$ is constrained by the size of the unit cell. Importantly, other design parameters can be modified to produce unit cells with high energy absorption e.g. ${\text{U}} = 14.2\,{\text{MPa}}$ is obtained from $\beta = \gamma = 153^\circ$ (Fig. 9c). This reinforces that $\gamma$ is a governing parameter that has a significant influence on the strain energy density of the bone-like unit cell. The unit cells in Fig. 9a,d that produce the peak strain energy density have relatively short tie lengths compared to the struts, which has the effect of increasing the critical buckling stress. The oblique struts of these unit cells produce more prominent flexural deformations, which consequently reduces the Young’s modulus and thereby increases the strain energy density according to Eq. (10).

## Conclusions

An analytical framework was developed to predict the critical buckling load, stiffness and energy absorption of a cellular structure based on trabecular bone. The framework was validated using experimental data obtained from compressive tests on a 3D printed bone-like cellular structure. The validated framework was used to run extensive simulations in parallel to obtain the most optimal unit cell configuration with high computational efficiency, using the energy absorption as the key performance criterion. It was observed that all design parameters, namely the sub-cell angles and tie lengths of the unit cell played a significant role in obtaining the highest energy absorption. Effectively, oblique sub-cell angles control the stiffness of the unit cell by balancing axial and flexural deformations. Specifically, unit cells with vertical column elements produced the highest stiffness whereas oblique unit cells exhibited a lower stiffness and higher energy absorption. The relative lengths of the columns and ties were found to control the buckling stress of the unit cell by modifying the joint stiffness of the column elements. It was observed that the optimal bioinspired structure showed re-entrant geometries in both sub-cells, which resulted in a low stiffness and high energy absorption. The optimal bioinspired structure was benchmarked against a re-entrant auxetic topology, which is extensively investigated in the literature, and showed an increase in energy absorption by a factor of four. The next step is to manufacture the optimal unit cells and assess their energy absorption capacity under static and dynamic loads. The analytical model will also be extended further to account for dynamic loading conditions. Machine learning techniques, including artificial neural networks and genetic algorithms, will also be employed in future work to reduce the number of simulations required under dynamic loading, which can be computationally expensive. The size effect of the unit cell can also be important under dynamic loading conditions and will also be investigated in future work.
